# Supplementary material for: Emergent User Behavior on Twitter Modelled by a Stochastic Differential Equation
Source: PLoS One. 2015 May 8;10(5):e0123876. doi: 10.1371/journal.pone.0123876 (PMC4425543; doi:10.1371/journal.pone.0123876)
Supplement: S1 Supporting Information — (PDF) [file pone.0123876.s001.pdf]

Information on data collection. The data used in the manuscript have been attached in one Tar GZip-compressed file directory in the Supporting Information S2 file “S2_dataset.tar.gz”. Each file included in the compressed directory contains the timestamps of the individual tweets in standard Unix time. The data was collected using the Twitter REST API. While data was also collected using the streaming API, we found no visible difference in the analysis of the two data sets. Below is a table of time period for the individual files.

Brand name	Start	End
BMW	Tue, 02 Oct 2012 19:10:35 GMT	Tue, 30 Oct 2012 10:30:26 GMT
Google	Mon, 17 Sep 2012 08:05:19 GMT	Mon, 15 Oct 2012 11:58:02 GMT
Gucci	Thu, 04 Oct 2012 11:47:54 GMT	Tue, 30 Oct 2012 10:25:49 GMT
Heineken	Fri, 24 Jan 2014 10:57:58 GMT	Thu, 27 Mar 2014 19:41:58 GMT
Pepsi	Wed, 10 Oct 2012 09:10:23 GMT	Tue, 30 Oct 2012 08:44:41 GMT
Samsung	Sun, 16 Sep 2012 09:44:11 GMT	Fri, 12 Oct 2012 07:47:12 GMT
Starbucks	Thu, 20 Sep 2012 07:23:39 GMT	Mon, 15 Oct 2012 12:09:09 GMT
